# Innovations in the veterinary intestinal health field: A patent landscape analysis

**DOI:** 10.1016/j.onehlt.2022.100419

**Published:** 2022-07-23

**Authors:** Marga E.M. Janse, Dick B. Zinkweg, Olaf F.A. Larsen, Linda van de Burgwal

**Affiliations:** VU Amsterdam, Athena Institute, De Boelelaan 1105, 1081 HV Amsterdam, the Netherlands

**Keywords:** Veterinary intestinal health, Probiotics, Enzymes, Prebiotics, Antibiotic use, One health

## Abstract

In recent years it has become evident that a healthy intestinal microbiome is beneficial for the overall health of an individual. A healthy microbiome is diverse, increasing stability and resilience and strengthening the immune system. In addition, healthy intestinal metabolisms have a beneficial effect on many physiological processes such as the brain function.

Looking from the One Health perspective, which recognizes that health of humans is closely connected to the health of animals and environment, it is inherently beneficial to stimulate the health of animals for the well-being of humans. However, the intensive administration of antibiotics to livestock for prevention and cure of disease, and even stimulation of growth, disrupts a healthy microbiome. With the rapid increase of emerging zoonotic diseases, alternatives to the use of antimicrobial compounds are urgently necessary. This research analyses the development of alternatives for antibiotic use contributing to veterinary intestinal health through an in-depth patent analysis of inventions for fodder additives. In the period 1999–2020, 1269 unique patent families describing the use of probiotics, enzymes and prebiotics for swine, poultry and ruminants were identified. Innovation trends, geography, key applicants, and classification of patents were analysed.

Asian industrial applicants applied for the majority of patents comprising the largest share of patents for probiotics and enzymes in combination with fodder for swine. Followed by North American and European industrial applications, applying for patents for probiotics in combination with fodder for poultry, swine, and ruminants. Overall, our results do not show a clear increase in innovations, suggesting that innovations in the use of probiotics and enzymes in animal feed appear to be stalling. While in the near future a combination of the use of antibiotics and alternatives is most likely to be implemented, the use of probiotics stands a good chance of replacing antibiotics in animal husbandry and limiting the adverse effects of antibiotic abuse.

## Highlights

• Alternatives for antibiotic use contributing to veterinary intestinal health are slowly coming to the market.
• Patent applications for probiotics, prebiotics, enzymes supporting a healthy veterinary intestine are stabilizing.
• China, as largest applicant for patents targeting the VIH,vcombines probiotics and enzymes with fodder for swine and poultry.

## 1 Introduction

In recent years it has become evident that a healthy intestinal microbiome is beneficial for the overall health of an individual. A healthy microbiome is diverse, thereby increasing stability and resilience [1], and strengthening the immune system. In addition, healthy intestinal metabolisms have a beneficial effect on many physiological processes such as the brain function [2]. The Human Microbiome research Project identified 2172 species residing in the human microbiome, with large inter-individual differences [3]. This project also confirmed that the microbiome in the Gastro-Intestinal-Tract (GIT) varies from human to human and is affected by an abundance of different factors making it an extremely complex system [4,5]. For modulation of the human intestinal health, evidence of using probiotics in treatment and prevention of disease is growing and new therapeutic areas for the use of probiotics are discovered [6]. An inherent hurdle to overcome here is the large inter-individual variation in gut microbiota composition (as well as temporal variation, so-called intra-individual variation), which poses challenges for the optimization of clinical trials [7].

The integrity of the human gut microbiome, however, should not be viewed in isolation of its surrounding ecosystems [8]. The animal ecosystem is of specific importance: the current human population consists of over 7.8 billion people [9], consuming more than 21 billion chickens, 1.4 billion cattle, 1.2 billion sheep, 1 billion goats and 900 million pigs in 2019, not including other livestock such as turkey and sheep [10].Together these five major livestock groups consist of over 26 billion animals used for consumption each year. The One Health approach states that it is beneficial to stimulate the health of animals for the well-being of humans [11]. However, the general administration of antibiotics to livestock for prevention and cure of disease, and even for stimulation of growth disrupts a healthy veterinary microbiome. This leads to dysbiosis and selection of antibiotic-resistant microorganisms [12], thereby adding to one of the top ten major global health threats as stated by the WHO [13].

In Europe, the increase of multi drug resistant (MDR) bacteria has led to a ban on the use of antibiotics for growth promotion, unlike in other meat-producing territories like e.g. the United States and Asian countries [14]. Across the globe, however, research has been carried out to find alternatives that support and promote Veterinary Intestinal Health (VIH) [15]. In recent literature a wide and varied range of alternatives is discussed: from carbohydrates preventing infectious diseases in the veterinary gut to, more recently, the administration of vaccines, antibodies, and probiotics [16]. Other alternatives include immune modulating agents, bacteriophages and their lysins, antimicrobial peptides, short-chain fatty acids, plant extracts, and inhibitors targeting pathogenicity; each stimulating the microbiome and the metabolism of the intestine [17].

Analogous to the results from the Human Microbiome Project, nutrition in general is a promising factor when it comes to improving VIH [18]. Combining food with live microorganisms like probiotics, is considered to have a positive effect on VIH by stimulating the growth of favorable bacteria in the GIT. For example, adding *Clostridia* and *Lactobacillus* bacteria to animal husbandry feed improved the efficiency of food uptake and consequently the growth of the animals [17]. Further alternatives to antimicrobial compounds for growth promotion are found in vitamins and enzymes, leading to a higher feed efficiency, supporting a better overall performance and having a positive effect on the immune system [19]. Whether these alternatives to antibiotics deliver on all their beneficial properties (promoting growth, and preventing and curing disease), however, remains to be seen.

Currently, it is unclear which of these alternatives are further developed in later-stage development, and which stakeholders are driving these developments. As innovation across life sciences and PharmaNutrition sectors critically depends on patenting as means to protect novel intellectual capital, patent documents can be used to signal these developments and identify stakeholders active in the field of VIH products/ additives [20]. While publication of inventive findings in patent documents can further stimulate innovations [21,22] and more fundamental research [23,24] in the VIH field, patents provide an abundance of information on geography, technology, and applicants [25]. Here, we perform a patent analysis for three frequently discussed VIH-approaches: probiotics, prebiotics, and enzymes, to obtain insight in the innovations in the VIH-field. As such, the main objective of this study is to provide insight into the current market of innovative products for VIH improvement by creating a patent landscape based on the analysis of patents for probiotics, prebiotics, and enzymes for three major livestock groups of poultry, pigs and ruminants [26].

## 2 Methods

### 2.1 Data collection

The patent family documents were acquired using the Espacenet databank (Worldwide. Espacenet), containing over 100 million patent documents from over 90 patent-granting authorities. Compared to other databases, Espacenet contains the best features for the search and selection of relevant patents [27] containing an abundance of VIH products that have the potential to reach the market.

#### 2.1.1 Search queries and classification codes

As the use of IPC and CPC codes within the eventual patent searches is more specific than only using keywords, the appropriate codes for the different animals and probiotics, prebiotics and enzymes were established thoroughly. First, a literature search was performed to get acquainted with the jargon of the VIH field and significant keywords were used to search relevant IPC/CPC codes. Table 1 shows an overview of the used keywords and the combinations with additional keywords deriving into the final patent dataset. Step by step the search query was expanded, and the resulting patents were assessed on relevance based on the title and abstract. Furthermore, deviating classification codes found in the final searches were additionally validated on relevance.

**Table 1:** Number of Patents after using the patent search syntax being the classification codes combined with key words for the period 1999  2020.

Classification codes	Initial search: “probiotics” or “prebiotics” or “enzymes”	Combined with ‘Intestine’ AND ‘Health’	Combined with ‘Fodder’ A23K50/75 A23K50/30 A23K50/10
Probiotics	Number of patents	Number of patents	Number of patents
A23L33/135 A23K10/18 A61K35/741 A61K2035/115	15.830	1206	789

Prebiotics			
A23L33/00 A23K20/00 A61K31/00 A61K36/00 A61K35/00	3.131	450	69

Enzymes			
C12N9/00 A23K20/189 A61K38/43	93.989	1362	411
Total		3018	1269

The search criteria were quality checked by an external expert on biomedical intellectual property from the Netherlands Enterprise Agency (RVO), a department of the Dutch Ministry of Economic Affairs. The search was executed in March–April 2021. Considering an 18-month publication delay, the publication date of all documents needed to be on or before 31st December 2019.

##### 2.1.1.1 Probiotics

Probiotics are defined as ‘live microorganisms which, when administered in adequate amounts, confer a health benefit on the host’ [28]. *Bifidobacterium* and *Lactobacillus* are examples of well-studied probiotic genera that can be used in foods or as supplements. For this study, we consider the overall benefit of probiotics in creating a more favorable gut environment by improving the quality of the gut microbiome [29].

The first search for patent documents was for the description codes of the use of probiotics for poultry, swine, or ruminant fodder and either one or more of the probiotic codes. Furthermore, the resulting patents should also have at least one or more of the words; intestin*, bowel* or gut (here no asterisk is needed, since no other endings of the word ‘gut’ exist implying the same meaning) in the title, abstract or claims specifying the search for fodder with the keyword fodder to search for “poultry feed”, “swine feed” and “ruminant feed” 789 patent family documents complied to these requirements and were used for further analysis.

##### 2.1.1.2 Prebiotics

Prebiotics are defined as ‘a substrate that is selectively utilized by host microorganisms conferring a health benefit’ [30]. Most established prebiotics are carbohydrate-based, next to other substances such as polyphenols and polyunsaturated fatty acids converted to respective conjugated fatty acids. The understanding of the health effects of prebiotics is evolving but foremost includes benefits to the gastrointestinal tract.

For the second patent document search, for prebiotics, again the search query was extended step-by-step. For the prebiotic search the animal fodder codes, keywords for intestines and the patent date requirement are the same as for the probiotic search. These codes were however less specific, for example the code A23K20 is described as a code for accessory food-factors for animal feeding-stuffs. As there were no extra specific classification codes found for prebiotics, the main classification classes remained “prebiotic*”. Specifying the search for fodder with the keyword fodder to search for “poultry feed”, “swine feed” and “ruminant feed” a total of 69 patent family documents were used for further analysis.

##### 2.1.1.3 Enzymes

Enzymes or feed enzymes are highly complex molecules catalyzing reactions in the GIT, leading to improved nutrient uptake and digestion due to various modes of action.

Furthermore, enzymes can be supportive for certain beneficial bacterial strains in the gut, thereby increasing the quality of the microbiome [31,32].

For the enzyme patent search also, a combination was made with the classification codes and the keywords. This final search for enzymes led to a total of 411 patents of use to improve the intestinal health of poultry, swine, and ruminants combined for fodder.

### 2.2 Data analysis

All patent documents were deduplicated based on patent family (i.e. multiple jurisdictional filings related to a single invention) and a description of the patent documents was downloaded in an Excel file for data analysis with Microsoft Excel. To understand the potential impact of inventions, the target markets of patented inventions were analysed. Given the high costs related to patenting, there is a direct relation between the location of patent applications and target markets. Patents are generally only applied for in countries where they are expected to bring a substantial economic benefit, either by generating revenues or by preventing competitors to enter the market [33]. Patent families for which only documents with kind codes belonging to the World Intellectual Property Organisation (WIPO) or European Patent Office (EPO) were categorized as a separate group. Analysis of the stakeholder type enhances the interpretation of the relevance of the data for the target market. To this purpose, unique patent applicants were identified, and patent applicants were categorized as Academia, Industry and Government as key applicants. Lastly the geographical information and innovative trend of patents for the three products was analysed and depicted in timelines.

## 3 Results

In total a final set of 1269 patents (see Table 1) were used for further analysis by assessing the innovation trends, geography, key applicants, and classification of the products.

### 3.1 Innovation trends

**Fig. 1:**
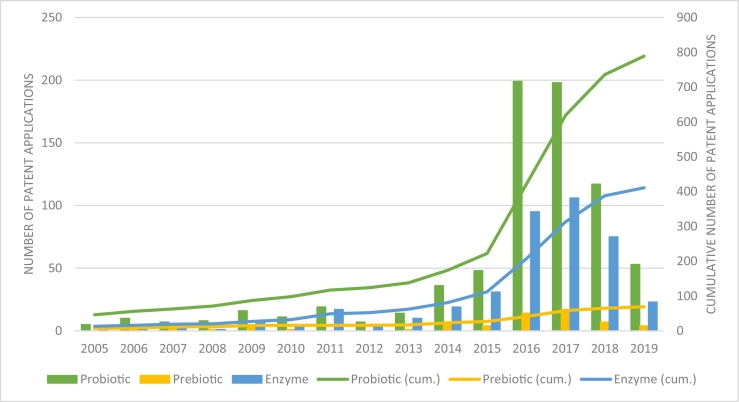
Stagnation of probiotic, prebiotic and enzyme patent applications after a clear increase in the time-period 2015–2018. The bars indicate the number of patents per year for pro-, prebiotics and enzymes for the combined group of poultry, swine, and ruminants. The lines indicate the cumulative number of patent applications.

In Fig. 1 the overall application trends of the patent families for Probiotics, Prebiotics and Enzymes are shown as a combined set for poultry, swine, and ruminants for the period 2005 until 2020. However scarce, patents have been filed before 2005: the earliest application date for probiotics was in 1968, for prebiotics 2000 and for enzymes 1986 leading to a starting point for the cumulative line in the graph of 46 patents applied for in the period 1999–2005. From 2010 onwards, a considerable growth for patent applications for enzymes and probiotics is seen, reaching its peak in 2016 and 2017. In 2016, the largest number of probiotics patents (199) were filed. In 2017, the largest number of prebiotic (17) and enzyme patents (106) were filed. After 2017, stagnation of the cumulative number of all three patent categories is shown.

### 3.2 Geographical distribution of the applications

**Fig. 2:**
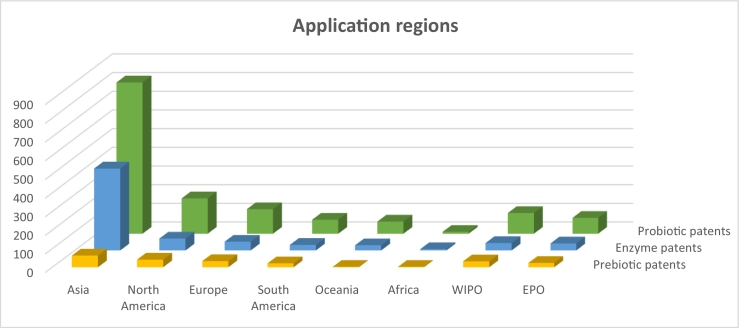
Asia is the largest designated territory for patent applications. The bars represent the continental distribution for the application of patents for probiotics, prebiotics, and enzymes for the combined livestock groups of poultry, swine, and ruminants.

The geographical distribution of the total of patent applications for probiotics, prebiotics, and enzymes for the combined group of poultry, swine, and ruminants is shown in Fig. 2. Worldwide, a significant portion of all patents are applied for in Asia, 48% of all probiotic patents, 21% of prebiotic patents and for 54% of enzyme patents are filed in this region. Within Asia, China is the prime target country, with 87% of all Asian probiotic's patents filed in this region, 76% of prebiotics and 88% of enzyme patents. China is followed by North America as second largest region for which patent applications are filed, constituting 7% share in the probiotic field, 10% of prebiotics and 5% of enzyme patents.

The least patent applications are filed in countries of the African continent, with South-Africa constituting 100% of those applications. The patent applications at the World Intellectual Property Organisation (WIPO) and the European Patent Office (EPO) show patents that are in first instance applied for in over 193 countries worldwide and for 38 member states in Europe. In later stages of the patent examination process, applicants can designate for which specific countries they want to claim patent rights. For this study, we did not further analyze this designation, which means that there is a possible overlap or double counting of patents between the assessed regions. At the EAPO (Eurasia Patent Organisation) only a few patents (*n* = 4) were filed, and these were excluded for further analysis. The probiotic patents applied for at the WIPO constitute approximately 8% of all the probiotic patents applied. For prebiotics this share is approximately 14% and for enzymes it is a 6% share.

**Fig. 3:**
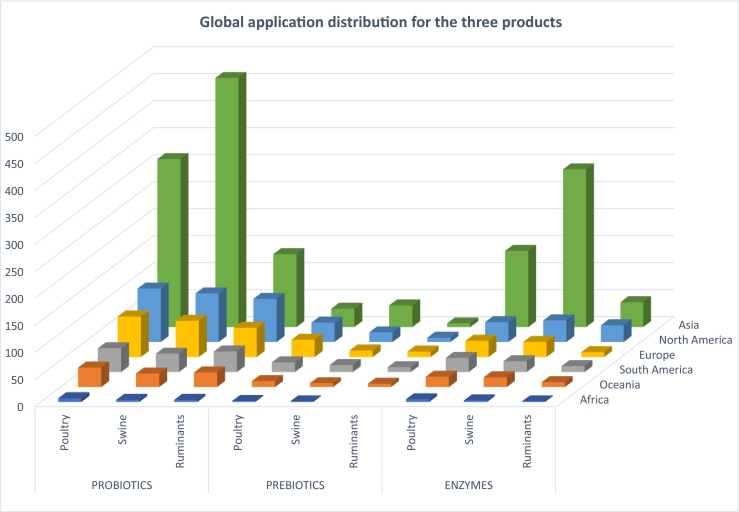
The majority of patent applications in Asia (predominantly China) are for probiotics and enzymes to be applied in all livestock categories, with most applications for swine. Prebiotic patent applications are spread over all regions, showing most applications for poultry for North America and for swine for Asia. The bars represent the number of patent applications in different continents separately for poultry, swine, and ruminants.

Further analysis of the patent data for probiotics, prebiotics and enzymes based on the separate livestock animal groups shows that most patent applications in Asia (China) are for probiotics and enzymes for swine and poultry (see Fig. 3). In North America and Europe, the relative differences in number of patent applications for interventions in poultry, swine and ruminant animals for probiotics and enzymes are much smaller. Applications for prebiotics patents are less prominent compared to probiotic and enzyme patents except for prebiotics for swine in Asia.

### 3.3 Applicants

**Fig. 4:**
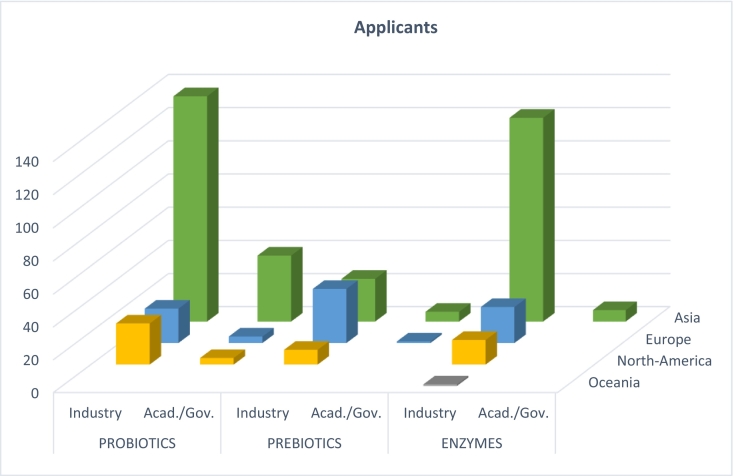
For both the probiotic and enzyme patents the Asian industrial and academic applicants dominate the field. European applicants from industry mainly apply for prebiotics patents. The bars depict the continental distribution of the origin of applicants for pro-, prebiotics and enzymes in the industrial, combined academic-governmental field.

Looking at the origin of applicants within the different fields, Asian industrial applicants applied for the majority of patents comprising the largest share of patents for probiotics and enzymes, respectively 69% and 71%. In contrast, most prebiotic patents are held by European industrial applicants, though differences are small in absolute terms (33 European industrial applicants vs. 26 Chinese industrial applicants). Overall, industry applicants were most prolific in all three application fields (prebiotics, probiotics, enzymes). Non or little applicants from government and/or academia, except for Chinese academia, were found applying for probiotic patents (See Fig. 4). No South American or African applicants were found for any of the three product types. Additionally, only one industrial Oceanian applicant, Anatara Lifesciences Ltd., was found applying for one patent involving enzymes.

### 3.4 Key applicant organisations

**Fig. 5:**
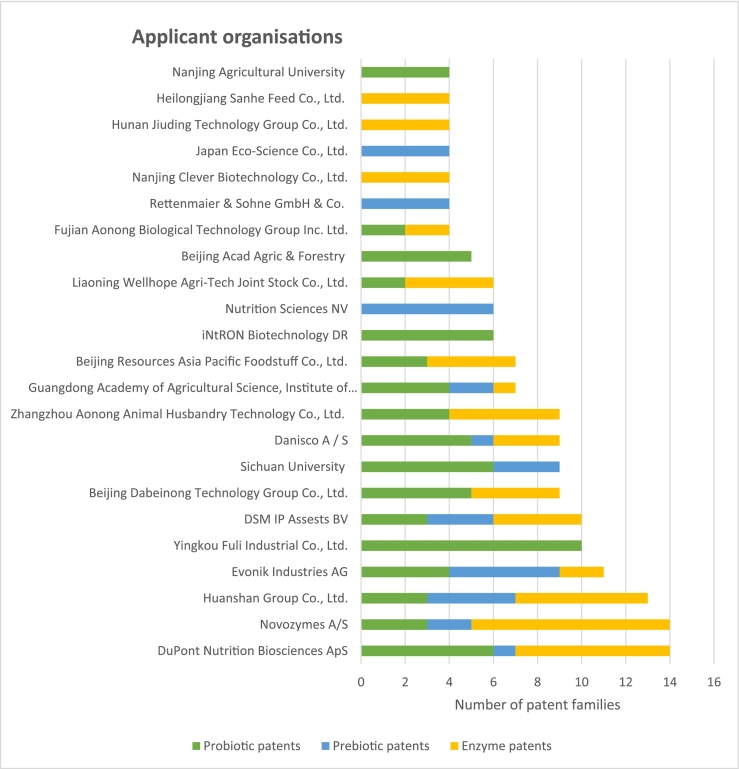
DuPont and Novozymes are key applicants for patents for all three product types. Novozymes is the applicant with most patents for enzymes, Yingkou Fuli Industrial Co., Ltd. is the applicant with most patents for probiotics and Nutrition Sciences NV is the applicant with most patents for prebiotics. Cut-off point is a minimum total of four patents for the three product types.

Looking more in-depth at the key applicant organisations from industry most of the applicants are from Asia and more specific of Chinese origin, next to a small group of non-Chinese key applicants (See Fig. 5). Also, the key academic applicants are Chinese: Nanjing Agricultural University, Beijing Academy of Agriculture and Forestry Sciences, Guangdong Academy of Agricultural Sciences and Sichuan University. DuPont (US) and Novozymes (Denmark) are the two non-Chinese industrial key applicants owning most patents for all three products (both 14 patents). Novozymes also owns the most patents for enzymes. Followed by three other non-Asian key applicants: Evonik Industrial AG with 11 patents, DSM IP Assets BV with 10 patents and Danisco A/S with 9 patents. Nutrition Sciences NV, a Belgian company, possesses the most patents for prebiotics. A Chinese company, Yingkou Fuli Industrial Co.Ltd., however, owns the most patents for probiotics.

## 4 Discussion

Here we show that despite initial increasing interest in modification of veterinary intestinal health innovations, patent applications for probiotics, prebiotics, and enzymes enhancing or supporting a healthy working veterinary intestine are stabilizing. In North America and Europe, the patents applied for probiotics in combination with fodder are most prominent for poultry, swine, and ruminants but total numbers are significantly smaller than in Asia, and in particular China. For all regions and all three animal groups, there are considerably less patents on prebiotics innovations than probiotics and enzymes. China is the largest applicant for patents targeting the veterinary intestinal microbiome by combining probiotic and enzyme products with fodder specifically for swine and poultry. North America and Europe are the second largest applicants for probiotic and enzyme products combined with fodder for all three livestock groups.

While initially patent applications grew in number, the growth rate declined after peaking in 2017. A typical explanation for decreasing growth rates in patent applications is market saturation [34]. Since the use of microbiome-modifying interventions is not yet well established in the veterinary field [17,35,36], it seems, however, that other reasons may play a role here. This research shows that industry stakeholders have been driving patent applications in this field and refocusing of industry partners due to innovation barriers may be another reason for declining innovation efforts. Mechanisms that hinder innovators to contribute to the open access knowledge base should be considered in light of existing innovation systems, with incentives and motivational drivers playing a key role in determining actions [[37], [38], [39]]. In view of these considerations, the increasing dependence on trade secrets within the field of antimicrobial innovations, and considerations of mechanisms to appropriate tangible and intangible resources are of increasing importance for stakeholders across pharma-nutrition industries [25]. As such, innovators may be moving away from the use of patents in exchange for alternative modes of knowledge appropriation.

Most patents for prebiotics, probiotics, or enzymes to improve veterinary intestinal health describe an application in fodder rather than in medicinal products. The efficacy and safety of probiotics in humans are to date still under discussion, despite the promising results [40]. In contrast, the regulatory burden for probiotics, prebiotics, and enzymes as additives to fodder is much lower, which could contribute to market introduction of these innovations through this alternative route [24]. To date, little research has been conducted to show the effect of enzymes on the human intestinal health [16]. Combined with limitations of enzymes in terms of activity, stability, quality and activity during production more research is needed to develop valuable products that address unmet needs [17]. These technological innovation barriers may explain why enzymes are underrepresented as approach to improve veterinary intestinal health in patent literature. Next to this, several studies agreed that more defined research and a better conceptualization of targeted unmet needs are also needed to establish positive effects of the use of prebiotics [41,42].

Chinese applicants are the most prolific in applying for patents in Chinese territory. This may suggest that the Chinese market is of particular interest for innovations regarding interventions targeting the VI-microbiome. Our finding of an abundance of patent applications for swine in Asia in comparison to other continents suggests that Asian countries, especially China, produce a considerably larger amount of pork than other regions [43]. Other possible explanations derive from a more economical perspective on patenting activity. The Chinese economy is transitioning from a labour-focused economy towards a more technology driven, innovative market, and is characterized by growing urbanization. These trends have an increasing effect on patent activity per se [44,45]. Moreover, stimulating policies by the Chinese government have resulted in an increase in venture capital investments in the biotechnological and pharmaceutical industries. Combined with Chinese public funding for these industries, a growth has taken place in the pharmaceutical industry in China, now being one of the biggest in the world [46].

## 5 Conclusion

In 2017, Centers for Disease Control and Prevention, estimated that 75% of newly emerging infectious diseases originate from livestock and wildlife animals, known as zoonoses (CDC, 2017). However, safeguarding the public health does not only comprise understanding the microorganisms of the human ecosystem but also those of other “non-human’ ecosystem like animal, plants, water, and soil supporting and conserve ecosystem resilience [8,47]. In the coming years this resilience is going to be challenged by the global increase in the use of antimicrobials expecting to increase with 11,5% in 2030 on all continents although a slower increase or even decrease in antimicrobial use in China is expected [48]. Next to these trends and the rapid increase of emerging zoonotic diseases, with most recently the Covid-19 pandemic, alternatives to the use of antimicrobial compounds are urgently needed. Implementation of alternatives that enhance the veterinary microbiome and positively effect animal growth and health should be considered. While in the near future, a combination of antibiotics and alternatives is most likely to be implemented [17,49], the use of probiotics is gaining traction [29]. In China, as an alternative to antibiotics, traditional herb feed additives are found to improve the growth performance of pigs. This corresponds to meeting the growing demand for pig meat, a trend that coincides with the revision of dietary guidelines in which recommended meat consumption is reduced to 40–70 g/day – at about half the current level [50,51]. In Europe, the unmet need is most pressing due to the ban on the use of antibiotics in livestock, while in the US consumer preferences are driving the reduction of antibiotics [17,26].

The development of innovations contributing to veterinary intestinal health should therefore be considered as an approach to implement alternatives for the use of antibiotics in livestock. Overall, however, our results don't show a significant increase in innovations as reflected in patent applications suggesting that innovations in the use of probiotics and enzymes in fodder may be coming to a halt. In the meantime, although the use of probiotics stands a good chance of replacing antibiotics in animal husbandry, the advice to use antibiotics wisely and to set up a scientific monitoring system remains the current best and fastest way to limit the adverse effects of antibiotic misuse.

## 6 Limitations

This study has searched for patents to find trends in innovations being an alternative for antibiotics used in animals. The use of patents to identify innovation trends is useful in industries that primarily depend on patents as a means to appropriate intellectual property. Such analyses, however, come with two notable limitations. First, it must be noted, that patents describe inventions rather than innovations, and not all patents result in commercial projects. Second, innovators in the pharma-nutrition industry may increasingly depend on alternative, less costly means to appropriate intellectual property as regulatory agencies have been reluctant in awarding health or disease-reduction claims to food. As a consequence, it is likely that there exist nutrition-based applications to support the GIT are in development while not covered in this study [52]. Nevertheless, the patent database provides a clear index of inventive activity in the field with the aim to further develop innovative products [25,27,33] and is as such useful for trend analyses This study is therefore a first attempt to map out innovation trend to identify potential alternatives to the use of antibiotics in the field of veterinary intestinal health.

## Funding

The contribution of LB is financed by the project Preparedness for Emerging Infectious Diseases with project number VI.Veni.201S.044 of the research programme Veni SGW which is financed by the Dutch Research Council (NWO). The funding source had no involvement in study design, data collection, data analysis, interpretation of the data, writing of the report or the decision to submit the article for publication.

## Author statement

All authors contributed to the revision of the original manuscript and agreed to send this revised final version.

## Declaration of Competing Interest

OL is also Senior Manager Science at Yakult. LB is consultant for several commercial parties in the field of probiotics and life sciences; none of her advising practices are in conflict with the content of this research.

## Appendix A Supplementary data

Supplementary material: IPC codes for fodder and medical use Image 1
