# Supplementary material for: Innovations in the veterinary intestinal health field: A patent landscape analysis
Source: One Health. 2022 Jul 23;15:100419. doi: 10.1016/j.onehlt.2022.100419 (PMC9582549; doi:10.1016/j.onehlt.2022.100419)
Supplement: Supplementary file 1 — Supplementary material: IPC codes for fodder and medical use [file mmc1.docx]

**Extra Material**

### Classification of the products

The most prevalent IPC codes describing all the different product types were classified. IPC codes were included when 10% of the patents and at least 10 patents included the class for minimal one product type. Three most used IPC codes for all three product types are all representing fodder applications and significantly more common than the IPC codes for medical preparations. This overview shows that probiotics, prebiotics, and enzymes are clearly combined with fodder based on these main IPC codes.

Fig. 6. The IPC codes for fodder (A23K10 and A23K20) are significantly more common compared to IPC codes intended for medical use (A61K35, A61K31 and A61P1).
